# Brain Networks Underlying Strategy Execution and Feedback Processing in an Efficient Functional Magnetic Resonance Imaging Neurofeedback Training Performed in a Parallel or a Serial Paradigm

**DOI:** 10.3389/fnhum.2021.645048

**Published:** 2021-05-25

**Authors:** Wan Ilma Dewiputri, Renate Schweizer, Tibor Auer

**Affiliations:** ^1^International Max Planck Research School for Neurosciences, Georg-August-University, Göttingen, Göttingen, Germany; ^2^Functional Imaging Laboratory, German Primate Center, Göttingen, Germany; ^3^Leibniz Science Campus Primate Cognition, Göttingen, Germany; ^4^School of Psychology, Faculty of Health and Medical Sciences, University of Surrey, Guildford, United Kingdom

**Keywords:** real-time neurofeedback, anterior midcingulate cortex, cortical and subcortical networks, learning, multitasking, reward processing

## Abstract

Neurofeedback (NF) is a complex learning scenario, as the task consists of trying out mental strategies while processing a feedback signal that signifies activation in the brain area to be self-regulated and acts as a potential reward signal. In an attempt to dissect these subcomponents, we obtained whole-brain networks associated with efficient self-regulation in two paradigms: parallel, where the task was performed concurrently, combining feedback with strategy execution; and serial, where the task was performed consecutively, separating feedback processing from strategy execution. Twenty participants attempted to control their anterior midcingulate cortex (aMCC) using functional magnetic resonance imaging (fMRI) NF in 18 sessions over 2 weeks, using cognitive and emotional mental strategies. We analyzed whole-brain fMRI activations in the NF training runs with the largest aMCC activation for the serial and parallel paradigms. The equal length of the strategy execution and the feedback processing periods in the serial paradigm allows a description of the two task subcomponents with equal power. The resulting activation maps were spatially correlated with functionally annotated intrinsic connectivity brain maps (BMs). Brain activation in the parallel condition correlates with the basal ganglia (BG) network, the cingulo-opercular network (CON), and the frontoparietal control network (FPCN); brain activation in the serial strategy execution condition with the default mode network (DMN), the FPCN, and the visual processing network; while brain activation in the serial feedback processing condition predominantly with the CON, the DMN, and the FPCN. Additional comparisons indicate that BG activation is characteristic to the parallel paradigm, while supramarginal gyrus (SMG) and superior temporal gyrus (STG) activations are characteristic to the serial paradigm. The multifaceted view of the subcomponents allows describing the cognitive processes associated with strategy execution and feedback processing independently in the serial feedback task and as combined processes in the multitasking scenario of the conventional parallel feedback task.

## Introduction

Neurofeedback (NF) is a psychophysiological technique that enables individuals to learn how to influence the activation of specific brain areas through executing mental strategies. It was developed as a therapeutic tool to enable patients to influence defined brain function, e.g., in clinical conditions associated with dysfunctional brain processes (Birbaumer et al., 2013; Linden, 2014). As brain activation cannot consciously be perceived, NF provides a perceivable feedback signal (usually visual), which reflects the activation of the targeted brain area or neural process (Sulzer et al., 2013). The trainee’s task (i.e., the NF task) is to find a mental strategy that influences this feedback signal in the expected way, thereby signifying the desired activation change in the targeted brain area. In the case of an efficient strategy, the feedback signal also acts as an operant conditioning reward, resulting in an increased likelihood of the trainees to re-execute the mental strategy.

NF was originally implemented using electroencephalography (EEG), which measures cortical neural activation at high temporal resolution. This provides the participants with immediate feedback on their efficiency in regulating the targeted brain area, as the two subcomponents of the NF task—execution of a strategy and processing of the resulting feedback—occur essentially simultaneously. The close temporal relationship between executing a strategy and receiving feedback, the high temporal resolution, and the relative convenience in practice made EEG NF a favorite tool not only for clinical therapeutic applications, i.e., stroke (Kober et al., 2017) or attention-deficit/hyperactivity disorder (Strehl et al., 2017), but also in the fast-growing field of brain computer interfaces (BCI) where a continuous signal in real time is essential, i.e., for the control of a robotic arm (Edelman et al., 2019).

Parallel to the EEG-based NF, the development of real-time functional magnetic resonance imaging (fMRI; Posse et al., 2003; Weiskopf et al., 2003) providing a blood oxygen level-dependent (BOLD) estimate within the MR image acquisition time usually within 2 s made fMRI feasible for NF. This is despite the 6-s latency of the BOLD response, which, together with the image acquisition and the real-time analysis, can delay the fMRI feedback signal, relative to the execution of the strategy by up to 10 s. In an rt-fMRI NF design, this is usually accounted for by a longer NF period. The drawback of the low temporal resolution and the BOLD latency in rt-fMRI NF is counterbalanced by the better spatial resolution and especially by the availability of whole brain coverage, providing robust signal not only from cortical but also from subcortical areas. This allowed to explore the feasibility of successful self-regulation (Sulzer et al., 2013; Sitaram et al., 2017), but moreover, it adds a supplementary view on NF: the description of brain areas and networks associated with the accomplishment of a NF training, i.e., of the “learning to self-regulate” (Haller et al., 2013; Emmert et al., 2016; Auer et al., 2018). Meta-analyses of whole-brain activations associated with the NF task have already described several brain areas as essential elements of the “neural substrates of self-regulation” independent of the targeted brain areas and executed strategies (Emmert et al., 2016; Sitaram et al., 2017).

Our present work supplements this description of brain areas and networks associated with the performance of the NF task by explicitly considering its subcomponents: strategy execution and feedback processing, performed in parallel as well as separately. The separation of the subcomponents was accomplished in a novel serial NF task design that complements the conventional parallel NF task. We implemented both designs in an fMRI-based NF study targeting the anterior midcingulate cortex (aMCC), a brain area generally involved in cognitive control (Shackman et al., 2011), to investigate the requirements for a successful NF training targeting a cognitive brain area (Dewiputri et al., 2013). The approach included shorter NF periods based on the expectations that cognitive strategies, being briefer in duration than emotional or motor imagery, would be applied to regulate the aMCC.

The parallel NF task follows the layout of a conventional NF of executing a strategy and perceiving the delayed feedback signal at the same time. Several mental processes have to be performed: the execution of a strategy, the perception and interpretation of the feedback signal, and the consideration of the feedback signal delay. The novel, serial NF task divides the NF task into distinct, successive periods of strategy execution without feedback followed by a period of feedback processing only with the same length as the strategy execution period, explicitly taking the latency of the NF signal into account. The feedback processing period thereby reflects the strategy execution period in full length. This allows to analyze and to describe the brain areas and networks associated with strategy execution and feedback processing as two separate subcomponents.

The present data analysis is based on the most efficient training run (mETR) of each participant, i.e., the run with the largest increase in the feedback signal. This outcome-oriented choice was made to limit the variability of the applied strategies to the most efficient ones. It also ensured that the feedback signal had a rewarding connotation and that participants through positive reinforcement will most likely choose the same strategy again without involving cognitive processes reflecting considerations on the next strategy execution.

Ultimately, our approach allows the description of brain areas and networks associated with efficient self-regulation from different viewpoints: (i) the multi-tasking perspective of the parallel condition, where strategy execution and feedback processing occur at the same time; and (ii) the single-task perspectives of separated strategy execution and feedback processing. The additional spatial correlation of the resulting activation maps with functionally annotated intrinsic connectivity brain maps (BMs) then permits insight into the underlying functional processes associated with the single subcomponents, as well as with the multitasking combination, and allows a comparison on the functional level. The direct comparison of the brain activation elicited during the NF in the parallel condition and the serial condition can further support the descriptive comparison and give some insight into the difference between multi-tasking vs. sequential single tasking.

## Materials and Methods

### Experimental Procedure

Twenty healthy, right-handed volunteers [8 males and 12 females; age, mean (SD): 25.9 years (5.3)] participated in the study. All participants provided written informed consent and received compensation for their participation. The study was approved by the local ethics committee of Georg-Ellias-Müller-Institute for Psychology at the University of Göttingen, Germany. Participants were randomly and equally assigned to either one of the two paradigms.

**Figure 1:**
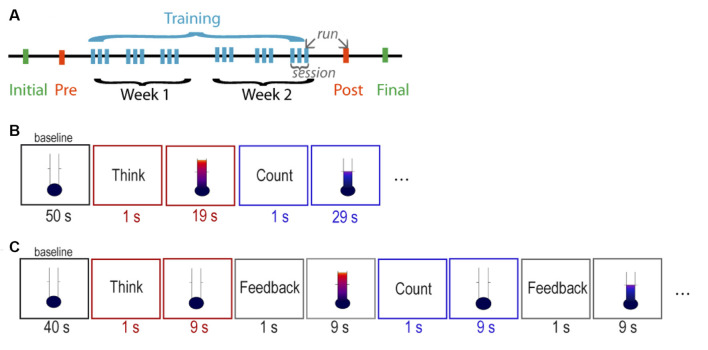
Structure of the neurofeedback (NF) training. **(A)** Overall structure of the NF study. Schedule of stimuli of the **(B)** parallel paradigm and the **(C)** serial paradigm.

The study consisted of 10 MRI sessions: one initial, one pre-training, six NF training, one post-training, and one final session (Figure 1A). In the initial and the final sessions, a high-resolution anatomical MRI scan was obtained. In the pre-training session, two functional measurements were obtained: (1) a functional localizer to determine the individual target region of interest (ROI) within the aMCC; and (2) a run of the assigned NF paradigm without receiving feedback (i.e., pre-training transfer). The functional localizer employed a continuous performance task (CPT; Heinrich et al., 2004) with the following parameters: trial length = 6,000 ms, presentation of visual stimulus (letter) = 250 ms, interstimulus interval = 5,750 ms, 80 trials, and total time = 8 min 12 s. The CPT was chosen because it is reported to elicit strong activation in the aMCC (Lütcke et al., 2009), as well as to be sensitive to NF training-related changes in this region (Gevensleben et al., 2014). The NF target ROI was identified individually based on the first-level activation map as the largest cluster within the aMCC defined by the Harvard–Oxford cortical structural probabilistic atlas thresholded at *P* = 0.25. Six NF training sessions were conducted on alternate days within 2 weeks, and each session consisted of three runs, in which the assigned NF training protocol was performed (a total of 18 training runs). The post-training session was scheduled 2 days after the last training session and consisted of: (1) one run of the assigned NF paradigm without receiving feedback (i.e., post-training transfer); and (2) the functional localizer task CPT.

### MRI Data Acquisition

All images were acquired on a 3T Tim Trio MRI scanner (Siemens Healthcare, Erlangen, Germany) using a 32-channel head coil for signal reception. Structural whole-brain T1-weighted images were obtained by an inversion-recovery 3D FLASH sequence (TR = 2,530 ms, TE = 3.26 ms, flip angle = 7°, TI = 1,100 ms, and 192 slices per slab) at 1.0 × 1.0 × 1.0 mm^3^ isotropic resolution. All BOLD fMRI measurements were obtained with a T2*-weighted gradient-echo EPI sequence (TR = 2,000 ms, TE = 36 ms, flip angle = 70°, acquisition matrix = 96 × 96) at 2.0 × 2.0 × 4.0 mm^3^ resolution with 22 slices oriented along the AC-PC line, encompassing the brain from the top to the level of the mid-brain. Individual slice positions from the first fMRI scanning session were subsequently re-applied in the following sessions (AutoAlign Scout, Siemens Healthcare, Erlangen, Germany) to minimize slice positioning differences. Motion correction was performed in k-space (online software of the manufacturer). An additional single EPI image with the same spatial resolution, but with 36 slices to obtain whole-brain coverage (TR = 3,250 ms, TE = 36 ms, flip angle = 70°), was obtained to optimize the registration of the partial-brain fMRI scan to the structural whole-brain MRI.

### Neurofeedback Training Paradigms

Visual feedback and instructions were given against a white background on a projection screen in the scanner bore. Visual feedback was presented as a vertical fluctuating thermometer scale, consisting of 21 feedback levels as gradations of color changing from blue (deactivation) to red (activation) with increasing height of the thermometer column. The feedback signal was updated at every TR (2 s). Independent of the paradigm, all participants were instructed to perform a task for two different periods within the run. For the periods starting with the word “THINK” projected on the screen, participants were instructed to use mental strategies that would increase the feedback signal. Suggestions for strategies were given in the domain of cognitive control (e.g., making plans and decisions) and emotions (e.g., think about a negative situation; Shackman et al., 2011). Participants were advised to keep a strategy constant within one period but were also encouraged to try various strategies across the periods. For the period starting with the word “COUNT,” participants were instructed to execute the strategy of covert backward-counting, with the goal to decrease the feedback level. If the attempted strategy did not result in a decrease, participants were free to search for another mental strategy to achieve the instructed effect.

#### Parallel Paradigm

The parallel paradigm started with an initial baseline period of 50 s, followed by six cycles of a 20-s “think” period alternating with a 30-s “count” control period, and ended with a 20-s baseline period (Figure 1B). During the 20-s “think” periods, in which the strategy was executed, continuous feedback was given in parallel. Similarly, during the 30-s “count” periods, continuous feedback was given during the execution of the strategy. The total length of the run was 6 min 17 s (185 images). Participants were informed about the 10-s intrinsic delay of the feedback signal.

#### Serial Paradigm

The serial paradigm started with an initial baseline period of 40 s, followed by eight cycles of a 10-s “think” strategy execution-only period, 10-s “feedback think,” 10-s “count” control period, 10-s “feedback count,” and ended with a 10-s baseline period (Figure 1C). The separation of the two subcomponents of NF task was implemented to consider the 10-s intrinsic delay of the feedback signal in the simplest possible way. Participants were instructed to try out a strategy for increasing the feedback signal during the 10-s “think” period, with no feedback during strategy execution, but receiving the feedback immediately afterwards in the 10-s “feedback think” period. The same principle was applied for the 10-s “count” period, in which the participants covertly counted backwards, with no feedback during strategy execution, but receiving feedback immediately afterwards during the 10-s “feedback count” period. The total length of the run was 6 min 17 s (185 images), identical to the parallel paradigm to ensure a similar participant fatigue level and compliance, as well as statistical power.

### Real-Time fMRI Neurofeedback

A custom in-house rt-fMRI NF system for rt-fMRI analysis and feedback presentation in MATLAB (The MathWorks, Inc., USA) was implemented (Dewiputri and Auer, 2013; Auer et al., 2015). Real-time data export from the MRI scanner via FTP allowed online fMRI analysis. Motion correction with an optimized SPM Realign algorithm^1^ was performed as a pre-processing step using the first volume of the localizer run as a reference.

The control ROI (bg) was the whole-brain mask; it was used to cancel out any global unspecific BOLD activation changes, e.g., general changes in blood flow or respiration. For both the aMCC target (aMCC) and the background control ROI, a percent signal change (PSC) was calculated for each time point (*t*), with reference to the average of five time points of the preceding “feedback count” period in the serial and 10 time points of the preceding “count” period in the parallel paradigm, respectively (bas). The feedback signal given to the participants was the difference between the PSC of the aMCC and the background, calculated as follows:

(1) $$FS_{t}=\frac{aMCC_{t}-aMCC_{bas}}{aMCC_{bas}}\%-\frac{bg_{t}-bg_{bas}}{bg_{bas}}\%$$

### Training Efficiency

Although we have collected a large dataset to assess and optimize the feasibility of aMCC regulation, we have decided to first investigate the effect of regulation at its maximum. This approach provides an initial proof of principle, as well as an opportunity to examine the underlying neural processes with great detail. Therefore, for each individual, the NF run with the highest training efficiency (mETR) was selected for analysis, to maximize the neural signal underlying the efficient self-regulation and to minimize the within-subject heterogeneity.

To assess the efficiency of the aMCC regulation, the BOLD signal (i.e., raw fMRI data) from the aMCC and the background region was extracted and analyzed offline using MATLAB. A general linear model (GLM) was performed on the extracted time courses with regressors representing the “think” period in the parallel paradigm or “think” and “count” periods in the serial paradigm. The GLM contrast was “think” for the parallel and “think” > “count” for the serial paradigms. The implicit baseline in the parallel paradigm corresponded to the “count” period; therefore, “think” for parallel can be interpreted as “think” > (baseline = “count”), which means that the contrast estimates from the two paradigms were comparable. Contrast estimates were converted to PSC, for the aMCC (PSC_aMCC_) and the background (PSC_bg_). The training efficiency TE was defined as the difference between the PSC_aMCC_ and the PSC_bg_ such that:

(2) $$TE=PSC_{aMCC}-PSC_{bg}$$

This estimate matches that of the feedback signal (see “Real-Time fMRI Neurofeedback” section) as close as possible while considering the whole data, thus providing a robust assessment of the overall feedback performance during the corresponding run.

The two paradigms were compared in terms of differences in NF training efficiency and in the timing of the selected mETR. Since efficiency values showed a normal distribution (*W* = 0.9379, *P* = 0.2185), we used an independent-sample *t*-test. The position of the mETRs among the 18 training runs showed a non-normal distribution (*W* = 0.8747, *P* = 0.0142), so the Mann–Whitney *U* test was used to compare run numbers across the two groups. Statistical analyses were performed with R version 3.6.1 (2019-07-05).

### Whole-Brain fMRI GLM Analyses

Whole-brain analysis of the fMRI data was conducted using FSL 6.0.1^2^. Data pre-processing included motion correction, brain extraction, spatial smoothing using a Gaussian kernel of FWHM 8 mm, and high-pass filtering with a cut-off frequency of 0.01 Hz. Registration of the partial fMRI volume to the whole-brain volume, and subsequently to the anatomical T1-weighted image, was performed using FLIRT in FSL, followed by non-linear registration (FNIRT) of the anatomical T1-weighted image to the standard MNI space. Individual scan-to-scan displacement time courses have been correlated with the time course of the events, and participants showing high correlations (larger than *Q*_1_ + 1.5 × IQR) have been excluded from the analysis. Of the 20 participants that completed the training, one subject from the parallel group was excluded from subsequent analyses due to excessive head motion.

First-level GLM models included regressors of interest accounting for “count” and “think” periods for strategy execution in both paradigms, as well as for “feedback think” and “feedback count” periods (combined “feedback” regressor) for feedback processing in the serial paradigm. Repetitions of “think” periods (six for parallel and eight for serial) were modeled separately using the least-squares all approach to take the inherently high trial-by-trial variability into account (Abdulrahman and Henson, 2016). Regressors were convolved with canonical double-gamma hemodynamic response function, and the models were completed with temporal derivatives of the regressors of interest, as well as the standard and the extended motion parameters. First-level contrasts were as follows: “think” for the parallel and “think” or “feedback” (i.e., two contrasts) for the serial paradigm. An overall contrast (i.e., “think” + “feedback”) was also generated for the serial paradigm when the two conditions have been considered equally. All contrast included “count” as a control condition (i.e., with a weight of −1). Regressor weights have been scaled so that all positive weights summed to 1. First-level contrasts were entered into higher-level analyses, modeling each group’s mean with a one-sample *t*-test using mixed-effects modeling in FSL (FLAME 1). *Z* (Gaussianized *T*) statistic images were thresholded using clusters of *Z* > 3.1 and a cluster significance threshold of *P* = 0.05 FWE corrected for multiple comparisons.

For an estimation of the overlap between the brain regions activated during the two paradigms, a conjunction analysis (Nichols et al., 2005) using the parallel “think” and the serial overall contrasts was performed. The resulting *Z* (Gaussianized *T*) statistic image was thresholded using clusters of *Z* > 2.3 and a cluster significance threshold of *P* = 0.01 FWE corrected for multiple comparisons (as defaults). To investigate which brain regions exhibit paradigm-specific activity, we also performed disjunction analyses (Robertson et al., 2015) by applying an exclusive mask of the resulting conjunction activity on each of the parallel and the serial overall statistic images. The resulting statistic images were thresholded using clusters of *Z* > 3.1 and a cluster significance threshold of *P* = 0.05 FWE corrected for multiple comparisons.

#### Functional Labeling

To provide a higher-level interpretation of the group-level activation maps, their correspondence with well-defined functional brain networks (Laird et al., 2011) was estimated. We used the *ICN*_Atlas toolbox (Kozák et al., 2017) to estimate the spatial correlation between the resulting group-level activation maps and the 20 intrinsic connectivity networks (ICNs) of the BrainMap database (Laird et al., 2011). The spatial correlation was chosen as a similarity measure because it takes into account both the activation extent and level, as well as its spatial variation.

#### Structural Labeling

Group activation maps were parcellated according to the Harvard–Oxford cortical and subcortical structural probabilistic atlas thresholded at *P* = 0.25. Results of the parcellation were overlaid on the MNI152 standard brain image. Regions were labelled according to whether the particular region was activated only in the parallel (red) or the serial overall contrasts (blue), resulting from the disjunction analyses, or activated in both paradigms as indicated by the conjunction analysis (green).

## Results

The present analyses identify and compare the brain areas associated with a NF task targeting the aMCC in two distinct paradigms that differed in the timing of feedback presentation: the parallel paradigm, which entails processing the delayed feedback concurrently with executing a mental strategy, and the serial paradigm, which temporally separates the strategy execution and feedback processing. We performed whole-brain fMRI analysis of the most efficient NF training (mETR), i.e., the training run with the largest increase of aMCC activation and positive feedback, separately for the three different conditions to obtain the associated BOLD-activation maps: (1) parallel paradigm: concurrent strategy execution and feedback processing; (2) serial paradigm: only strategy execution; and (3) serial paradigm: only feedback processing. These maps were further correlated with ICNs associated with specific functions to gain insight into the processes associated with the activated brain areas and the performed tasks.

### Efficient Neurofeedback Training Runs

**Figure 2:**
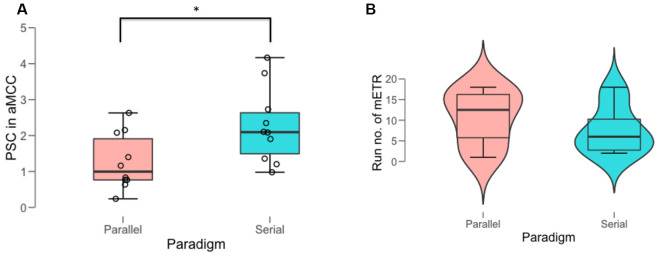
Behavioral outcomes of the most efficient training run (mETR). **(A)** Percent signal change (PSC) in anterior midcingulate cortex (aMCC) achieved during the mETR in the two paradigms. **(B)** Distribution of the temporal position of the mETR (run no. of mETR) within the schedule of the 18 NF training runs in the two paradigms. *Denotes significant difference at the threshold of *p* = 0.05.

Participants in the serial paradigm performed the NF tasks more efficiently compared to those in the parallel paradigm, evident by a significantly larger training efficiency, i.e., increase of the BOLD amplitude in the aMCC relative to that in the whole brain [mean (SD): 2.3 (1.0) vs. 1.27 (0.8); *t* = −2.4, df = 16.665, *P* = 0.028] during the most efficient NF training run (mETR; Figure 2A, Table 1). The analysis of the distribution of the mETR showed that the mETR occurred at various stages during the NF training (Table 1), with no significant difference (*W* = 43.5, *P* = 0.65) between the serial paradigm (median: 6) and the parallel paradigm (median: 12.5; Figure 2B).

**Table 1:** The run number, i.e., position, of the most efficient training run (mETR) within the 18 training runs and its corresponding training efficiency (TE) in percent signal change (PSC), i.e., PSC_aMCC_ minus PSC_bg_ for each subject as well as their central tendency (median for mETR and mean for TE) in one of the two feedback paradigms.

Subject	mETR	TE (PSC)	Subject	mETR	TE (PSC)
P01	11	2.2	S01	8	2.7
P02	1	0.8	S02	18	3.7
P03	18	1.2	S03	11	2.1
P04	14	0.8	S04	6	1.2
P05	1	2.1	S05	2	1.9
P06	18	2.6	S06	2	2.1
P07	8	0.8	S07	5	1.4
P08	5	0.6	S08	6	2.3
P09	14	1.4	S09	16	1.0
P10	17	0.2	S10	2	4.2
**Median/mean**	**12.5**	**1.27**	**Median/mean**	**6**	**2.3**

*P, parallel paradigm; S, serial paradigm*.

### Whole-Brain fMRI Analysis of Efficient Neurofeedback Run

#### Brain Areas and Brain Networks Associated With Neurofeedback Subcomponents

**Figure 3:**
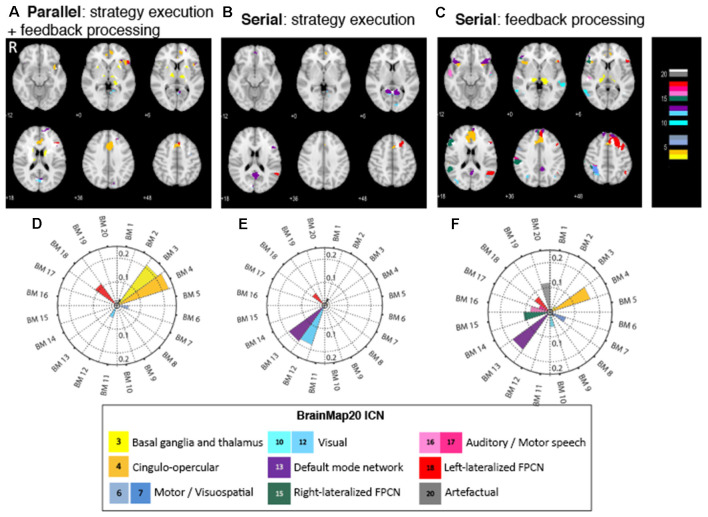
**(A–C)** Brain regions activated in the NF subcomponents across the three different conditions. Color code corresponds to the BrainMap20 (Laird et al., 2011) atlas base map.** (D–F)** Spatial correlation of brain activity in NF with the BrainMap20 intrinsic connectivity networks (ICNs). FPCN, frontoparietal control network. Thresholded and unthresholded activation maps are available at https://neurovault.org/collections/7730.

The parallel paradigm revealed activation in brain areas that were correlated to three brain networks (Figures 3A,D). The most prominent activation is of the anterior cingulate cortex (ACC), which can be related to the successful regulation of the aMCC—the target region within the ACC—by the executed strategy. The pattern of activations of the ACC, the anterior insula (aI), and the frontal cortex spatially correlates with BM 4 (*r* = 0.23), the cingulo-opercular network (CON). This network, also known as the salience network (Menon, 2011), is a transitional network linking cognition and emotion/interoception (Laird et al., 2011). The concurrent task also activated the basal ganglia (BG), with a pattern spatially correlating with BM 3 (*r* = 0.21), which is strongly associated with reward. The pattern of activation in the left superior, medial, and inferior frontal gyri spatially correlated with the left frontoparietal control network (L-FPCN) BM 18 (*r* = 0.11), which is associated with memory tasks. Additionally, there was a small spatial correlation with the activation pattern in the visual areas, BM 12 (*r* = 0.05).

The strategy execution component of the serial paradigm exhibited activation in fewer brain areas and was associated with fewer brain networks than that in the parallel paradigm (Figures 3B,E). Activation in the ACC again points to successful strategies activating the targeted brain area. A set of areas that consists of the precuneus, cuneus, posterior cingulate cortex (PCC), and frontal medial cortex (FMC) was prominently activated, and the pattern of their activity spatially correlated with the default mode network (DMN), BM 13 (*r* = 0.19). The second set of brain activations, the lingual gyrus and calcarine cortex, is correlated with the visual network, BM 12 (*r* = 0.18). The third set—the left superior frontal gyrus (SFG) and left middle frontal gyrus (MFG)—collectively classified as the dorsolateral prefrontal cortex (DLPFC), is part of the left (FPCN), BM 18 (*r* = 0.06), and is associated with memory tasks.

The activation map of the feedback processing of the serial paradigm displays a wide range of brain areas associated with several brain networks (Figures 3C,F). ACC activation is prominent, which indicates a role of this area in feedback processing. Feedback processing activated brain areas associated with the DMN (BM 13; *r* = 0.19): FMC, precuneus, PCC, and inferior frontal gyrus (IFG). Also activated are brain areas associated with the CON (BM 4; *r* = 0.17): ACC, paracingulate gyrus, and bilateral aI. Two sets of brain areas are associated with the lateralized, left and right, frontoparietal control networks (R-FPCN, BM 15, *r* = 0.106 and L-FPCN, BM 18, *r* = 0.07): superior parietal lobule (SPL), supramarginal gyrus (SMG), angular gyrus (AG), and medial and superior frontal gyrus (MFG, SFG). Other areas that showed increased activation were the motor areas (BM 7), visual areas (BM 10), auditory areas (BM 16), as well as areas associated with spatial normalization artifacts (BM 20; Table 2).

**Table 2:** Prominent BrainMap20 intrinsic connectivity networks that are spatially correlated to activation maps in the neurofeedback subcomponents in the three conditions.

Neurofeedback subcomponent				
Parallel (strategy+ feedback)	Serial: strategy execution	Serial: feedback processing	BM20 atlas areas	Function (Laird et al., 2011), selected by relevance to NF task
–	BM2	–	sgACC and OFC	Emotion and a strong preference for a reward task.
BM 3	–	*	Bilateral BG and thalamus	Mostly strong preference for a reward task.
BM 4	–	BM 4	Bilateral AI and fO	Transitional network linking cognition and emotion or interoception.
BM 6	–	–	SFG and MFG including premotor and SMC	Action imagination, learning and recall of complex sequences.
–	–	BM 7	MFG and SPL	Visual processing, counting, and calculation.
–	–	BM 10	MTG and ITG	Viewing complex stimuli and mental rotation.
BM 12	BM 12	–	Medial POC	Simple visual stimuli.
–	BM 13	BM 13	MPFC, PCC, and precuneus	DMN, imagine scenes, and episodic memory.
–	–	BM 15	R-FPCN	Reasoning, attention, and memory.
–	–	BM 16	Transverse temporal gyri	Audition, music, and speech.
–	–	BM 17	Dorsal precentral gyri, central sulci, and postcentral gyri	Sensorimotor cortices for mouth region, associated with speech, reading, and swallowing.
BM 18	BM 18	BM 18	L-FPCN	Working memory and explicit memory tasks.
–	–	BM 20	Artefactual	Artefact from pre-processing.

BM, brain map; BG, basal ganglia; ACC, anterior cingulate cortex; AI, anterior insula; fO, frontal operculum; ITG, inferior temporal gyrus; L-FPCN, left-lateralized frontoparietal network; MFG, middle frontal gyrus; MPFC, medial prefrontal cortex; MTG, middle temporal gyrus; NF, neurofeedback; sgACC, subgenual ACC; SMC, somatomotor cortex; PCC, posterior cingulate cortex; POC, posterior occipital cortex; PreCu, precuneus; R-FPCN, right-lateralized frontoparietal networks; SFG, superior frontal gyrus; SPL, superior parietal lobule. *Only the thalamus is seen in BM3 of the feedback processing task.

#### Common and Specific Neurofeedback Networks

**Figure 4:**
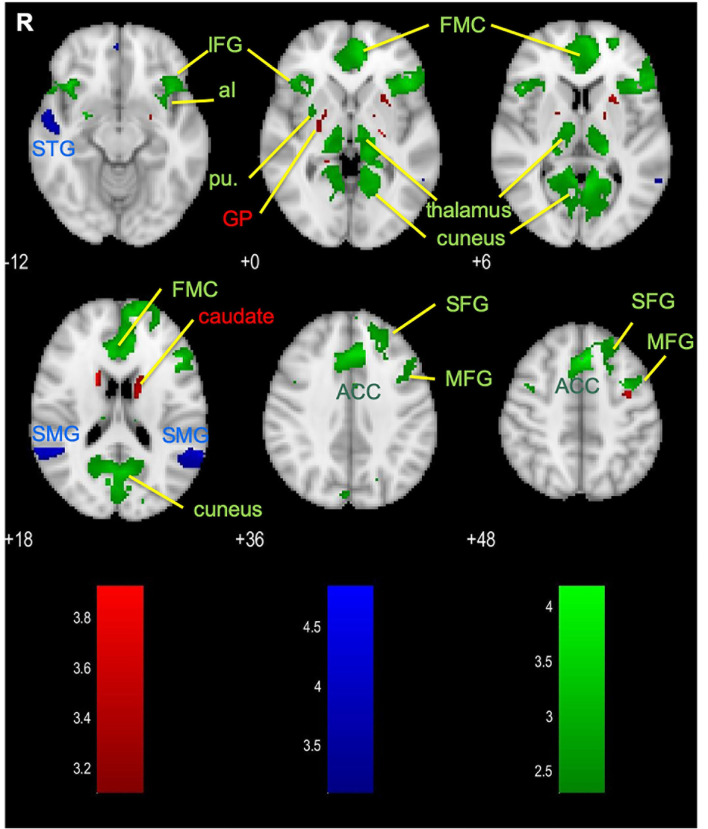
Brain areas that are dominantly activated in the disjunction of parallel paradigm (red), serial paradigm (blue), and the conjunction of the parallel and the serial paradigms (green). ACC, anterior cingulate cortex; aI, anterior insula; FMC, frontal medial cortex; GP, globus pallidus; IFG, inferior frontal gyrus; MFG, middle frontal gyrus; pu, putamen; SFG, superior frontal gyrus; SMG, supramarginal gyrus; STG, superior temporal gyrus.

To gain an overall picture of the networks that are commonly involved in executing a mental strategy concurrent with or separate from processing feedback, we combined the BMs associated with the parallel and the serial paradigms (Figure 4, green). The resulting set of brain areas comprises three major large-scale cognitive networks: the DMN (anchored in FMC, PCC, and precuneus), CON (anterior cingulate and bilateral aI), and FPCN (dLPFC and PPC). Additional brain areas such as the visual association areas, putamen of the BG, and the thalamus were activated.

We also asked the question if there are specific differences in brain activation associated with the temporal separation of the strategy execution and the feedback processing, i.e., if there are brain areas dominantly activated in either the parallel paradigm or the serial (strategy execution and feedback-processing period) paradigm. Our disjunction analysis revealed a small set of brain areas was activated more evidently during the concurrent strategy execution and delayed feedback processing in the parallel paradigm (Figure 4, red): the caudate, putamen, and globus pallidus (GP)—all of which make up the BG. The overall contrast of the serial paradigm revealed activations in the bilateral SMG and right superior temporal gyrus (STG; Figure 4, blue), which were found to be more evident for this paradigm. Since these brain areas are part of the activation map of the feedback period, they can most likely be attributed to the feedback processing (Figure 3B).

## Discussion

The aim of this work was to identify and compare the brain activation associated with strategy execution and feedback processing, performed concurrently in the parallel paradigms or separately in the serial paradigm, during the mETR. Participants of the serial paradigm were able to achieve higher aMCC activation than participants of the parallel paradigm. Whole-brain analysis across the three conditions showed that the pattern of brain activation in the parallel paradigm is spatially correlated (i.e., had a similar spatial distribution) with the BG network and the CON. Activations in the strategy execution task of the serial paradigm spatially correlated with the intrinsic activations of the visual-processing network, the DMN, and the FPCN, while activations during the feedback processing task spatially correlated with the intrinsic activations of the CON, the DMN, and the FPCN. Contrasting the activation elicited by the parallel paradigm with the combined activation of the serial strategy and serial feedback revealed that BG activation was dominantly specific to the parallel paradigm, while only the activations of the STG and SMG were dominantly specific to the serial paradigm.

### Training Efficiency

Self-regulation of the aMCC brain activity was more efficient when feedback processing was separated from strategy execution; this observation is also in line with other studies (Johnson et al., 2012; Emmert et al., 2017; Oblak et al., 2017; Hellrung et al., 2018). The temporal separation of strategy execution and feedback processing leads to two advantageous effects: (1) reduction in cognitive load; and (2) more intuitive link between behavior and reward. When following the parallel paradigm, participants have to multitask by executing a mental strategy, processing feedback, and estimating its timing. On the other hand, when following the serial paradigm, participants can focus on performing one subcomponent of the NF task at a time: first executing a mental strategy, then perceiving and processing the corresponding feedback. Separating the multitasking into a sequence of two consecutive tasks reduces the cognitive load. The second effect is associated with the characteristics of the delayed feedback signal and its temporal ambiguity. Missing time cues in the parallel paradigm cause a credit assignment problem (Oblak et al., 2017), namely, participants must recall which executed mental strategy caused the change in the feedback. The serial paradigm resolves the ambiguous temporal contingency by informing participants about the timing and increases the contiguity through the one-to-one relationship of the feedback to the strategy execution. Our observations are in line with other studies that showed awareness of the delay in the feedback can facilitate causal learning (Greville and Buehner, 2010; Greville et al., 2013). Therefore, we can state that the unambiguous temporal contingency and contiguity of the feedback in the serial paradigm facilitates operant learning in NF.

### Methodological Considerations

The difference in self-regulation achieved in the mETR in the serial compared to the parallel paradigm group triggers the discussion of the comparability of the two NF designs. Although the parallel paradigm used longer total regulation time (6 × 20 s = 120 s) in comparison with the serial (8 × 10 s = 80 s), the longer time is divided between the strategy execution and the feedback evaluation. We generally do not have data on how participants meet this multitasking challenge in the parallel paradigm, but we assume task switching between the two subcomponents because there is a logical order between them: i.e., the feedback evaluation informs the strategy execution. Based on the low temporal resolution and since the periods are modeled with conditions in their entirety, the resulting fMRI activations represent the process or processes dominantly being present during the given period. While in the parallel paradigm there is a single condition, which is considered to be an unknown mixture of the two subcomponents, the serial paradigm contains conditions representing the pure subcomponents.

An additional concern may be that the higher efficiency in the mETRs could be explained by a higher subject engagement and subsequent better data quality, which could ultimately lead to an overestimation of the overall effect, i.e., higher efficiency in both paradigms. However, the overall effect is orthogonal to the effect of interest, which is the difference between the paradigms.

### Brain Areas and Networks in the aMCC Neurofeedback Subcomponents

The identified brain areas and networks that are active during the concurrent NF subcomponents allow inferences about the cognitive processes involved and their interaction. The brain areas activated in the parallel NF paradigm are similar to the general findings of other NF studies. Specifically, the ACC, the aI, and the BG are brain areas associated with the neural mechanisms of NF-assisted self-regulation (Emmert et al., 2016; Sitaram et al., 2017).

One of the most prominent brain areas activated is the ACC, which can at least partially be attributed to the successful self-regulation of the aMCC, a smaller target region within the ACC. It is also a positive control considering our data selection approach based on the training efficiency. The ACC is functionally connected to the insula (Menon and Uddin, 2010; Shackman et al., 2011) and is part of the CON—a transitional network linking cognition and emotion or interoception (Laird et al., 2011), with which the activation pattern of the parallel paradigm is correlated. This network, also called the salience network, monitors the salience of external inputs and internal events (Bressler and Menon, 2010) and mediates the switching between internalized and externalized cognition (Sridharan et al., 2008; Bressler and Menon, 2010; Goulden et al., 2014). Activation of the CON reflects the multitasking demands of the parallel paradigm: participants need to execute an internal mental strategy while concurrently processing external feedback. Of course, the correspondence between the active brain regions/networks and the NF subcomponents cannot be confirmed in the parallel paradigm due to the lack of temporal separation.

The second prominently activated brain areas are the BG, which are associated with a network strongly activated by reward tasks. The involvement of the BG in NF learning is a well-established concept and has been shown in animals (Koralek et al., 2012) as well as in human studies (Birbaumer et al., 2013; Sitaram et al., 2017; Skottnik et al., 2019). Evidence for the importance of the BG also comes from a meta-analysis of fMRI NF studies, where the BG are consistently active during NF (Emmert et al., 2016). The BG are also targets of the midbrain dopaminergic neurons, which transmit phasic signals that convey a reward prediction error—a neurophysiological signal that relates to how unexpected or surprising an outcome (Walsh and Anderson, 2011). The reward prediction error is then transmitted to the ACC, which evaluates it and provides a signal for behavioral adjustment—whether to reinforce or to punish the actions preceding the outcome (Walsh and Anderson, 2011; Amiez et al., 2012). In the context of our NF experiment, activation of the caudate and the GP of the BG seen primarily in the parallel paradigm could be interpreted as evidence for fine-tuning of strategies according to the concurrent feedback that participants receive. Even though the BG activation is more evident in the parallel condition, the challenging multitasking condition with the concurrent strategy execution, estimating the feedback delay, and evaluating the feedback signal results in lower activation during the mETR than in the serial condition (Figure 2). However, since these results are based on a limited set of data, further analyses are necessary to reappraise this finding.

The separation of the two NF subcomponents in the serial paradigm allowed us to distinguish the brain areas and networks that potentially overlap in the parallel paradigm. The brain areas activated during strategy execution were the smallest and the least distributed. However, it must be acknowledged that the experimental design has less power to detect activation during strategy execution than during feedback evaluation due to the lower number of periods for the former. The distinct activation of aMCC most likely results from the successful mental strategy, as expected based on data selection. The involvement of the DLPFC and the posterior parietal cortex (PPC) in NF supports previous findings (Emmert et al., 2016), specifically those associating it with NF control (Sitaram et al., 2017). This is reflected in the correlated network, the FPCN, also known as the central executive network, which is engaged in higher-order cognitive and attention control, mediating goal-directed behavior through attention shifting (Bressler and Menon, 2010). The other network involved in strategy execution correlated with the cuneus and precuneus, i.e., the DMN (Bressler and Menon, 2010). This network is associated with internally focused behavior, such as self-referential processing (Buckner et al., 2008; Andrews-Hanna et al., 2010), and it thus reflects the assigned task: to perform a mental strategy to self-regulate the aMCC. These two networks distinctively represent the processes associated with finding and executing a mental strategy for self-regulation, i.e., the combination of externally oriented, goal-directed behavior and internal self-referential mental processing. FPCN can couple with DMN when a goal-directed behavior requires endogenous, self-referential thoughts (Dixon et al., 2018) or autobiographical planning (Spreng et al., 2010), and this FPCN–DMN coupling could be hypothesized to allocate resources toward executing an intrinsic cognitive process to increase the NF signal.

Brain areas activated specifically during feedback processing reveal a complex pattern of activation and are correlated with a larger number of brain networks, reflecting the complexity of the task itself. Three major cognitive networks are present in the delineated feedback processing subcomponent: the DMN, the FPCN, and the CON, which are also shown to be involved in the parallel paradigm (FPCN and CON) and the delineated strategy execution subcomponent (FPCN and DMN). It can be considered that the different aspects of the feedback processing are reflected in the activation of the different networks. The activation of both the DMN and the FPCN during the feedback period could reflect a recollection of the executed strategy during the feedback period. This is instrumental since the outcome of the feedback has to be related to the strategy performed. The involvement of the CON can also be associated with processing of the outcome of the feedback, i.e., relating the internal representation of the executed goal-directed strategy (DMN–FPCN) with the reward saliency of the external feedback signal (BG) similarly to what we have seen for the parallel paradigm.

The analysis of the feedback processing period also revealed the activation of the aMCC as part of the prominently activated ACC. This finding further clarifies the involvement of this brain area we have also seen in the parallel paradigm and supports earlier works suggesting the involvement of the aMCC/ACC in feedback processing in both EEG NF (Gevensleben et al., 2014) and fMRI NF (Auer et al., 2015), as well as in a trial-and-error problem-solving task (Amiez et al., 2012).

The overlap of the parallel and the combined (i.e., strategy and feedback) serial conditions further confirms the findings of the functional labeling and demonstrates the involvement of the DMN, FPCN, CON, and BG/thalamus in both paradigms (Figure 4, green). Contrasting the parallel paradigm activation with the combined serial conditions, we also explored whether brain areas are dominantly active during the multitasking parallel paradigm or specific to the serial paradigm. Similarly to what we have seen in functional labeling, the parallel paradigm showed a larger sensitivity in the BG (Figure 4, red). On the other hand, the activation of the STG and the superior medial gyrus (SMG) is more evident in the serial paradigm (Figure 4, blue), which reflects activation being present during the dedicated feedback-processing period. These two areas have been proposed to exert a top-down control of visual processing (Shapiro and Hillstrom, 2002), which relates to the higher-order cognitive influence on processing the visual information during the feedback evaluation period.

## Conclusions

Our results reveal that separating strategy execution from feedback processing is able to dissect the usually overlapping cognitive subcomponents of the NF tasks and to reveal insights into the interplay of the whole-brain networks involved. Although the inferences from the brain activation to the cognitive processes associated with the NF tasks generally concur with the existing knowledge, further studies and analysis on the contribution of the subcomponents to the task will paint a more comprehensive picture on how networks work together in NF to support these processes. This more general understanding of the NF task could further explain individual variances in NF performance and help optimize NF paradigms.

## Data Availability Statement

The datasets presented in this study can be found in online repositories. The names of the repository/repositories and accession number(s) can be found below: https://neurovault.org/collections/7730.

## Ethics Statement

The studies involving human participants were reviewed and approved by local ethics committee of Georg-Ellias-Müller-Institute for Psychology at the University of Göttingen, Germany. The patients/participants provided their written informed consent to participate in this study.

## Author Contributions

WD, TA, and RS conceptualized and designed the experiment and wrote and revised the manuscript. WD implemented the experiment. WD and TA analyzed the data. All authors contributed to the article and approved the submitted version.

## Conflict of Interest

The authors declare that the research was conducted in the absence of any commercial or financial relationships that could be construed as a potential conflict of interest.

## Footnotes

^1^http://www.fil.ion.ucl.ac.uk/spm/

^2^www.fmrib.ox.ac.uk/fsl
